# Extracellular Vesicles in Diffuse Midline Glioma: Emerging Mediators of Radiation Response and Therapeutic Resistance

**DOI:** 10.3390/cancers18121933

**Published:** 2026-06-13

**Authors:** Ann Morcos, Yeonkyu Jung, Ryan N. Fuller, Jessica M. S. Jutzy, Nathan R. Wall

**Affiliations:** 1 Department of Radiation Medicine, James M. Slater, MD Proton Treatment & Research Center, Loma Linda University Health, Loma Linda, CA 92350, USA; amorcos@llu.edu (A.M.); yeonkyu.jung@email.ucr.edu (Y.J.); jessicajutzy@llu.edu (J.M.S.J.); 2 Department of Pathology and Human Anatomy, Loma Linda University School of Medicine, Loma Linda, CA 92350, USA; 3 Department of Biological Sciences, California Baptist University, Riverside, CA 92504, USA; ryfuller@calbaptist.edu

**Keywords:** diffuse midline glioma, extracellular vesicles, exosomes, liquid biopsy, radiation response, radioresistance, tumor microenvironment

## Abstract

DMG, including DIPG, is a highly aggressive pediatric brain tumor with dismal clinical outcomes. Radiotherapy remains the cornerstone of treatment, yet responses are transient and resistance is nearly universal. Emerging evidence indicates that EVs are key mediators of radiation response, facilitating intercellular communication and the propagation of radioresistant phenotypes within the tumor microenvironment. EVs carry diverse molecular cargo, including RNAs, proteins, and lipids, that can dynamically influence tumor behavior and treatment response. In this review, we focus on the role of EVs in shaping radiation response in DMG, while also examining their broader functions in tumor biology, biomarker development, and therapeutic delivery. We summarize evidence for EV-mediated regulation of tumor growth, invasion, microenvironmental interactions, and immune modulation. We further discuss the potential of EVs as minimally invasive biomarkers for liquid biopsy, highlighting both their advantages and current limitations relative to circulating tumor DNA (ctDNA) approaches. In addition, we review emerging strategies utilizing EVs as therapeutic delivery platforms capable of crossing the blood–brain barrier (BBB) and delivering small molecules and nucleic acid-based therapies. Finally, we explore the role of EVs in modulating radiation response, including their contribution to radioresistance and their potential as biomarkers of treatment efficacy. Although EV-based approaches hold significant promise in DMG, challenges related to standardization, specificity, and clinical validation remain. Continued investigation into EV biology and translational applications may provide new opportunities for improving diagnosis, monitoring, and treatment of this devastating disease.

## Simple Summary

Diffuse midline glioma (DMG), including diffuse intrinsic pontine glioma (DIPG), is a devastating childhood brain tumor for which radiotherapy remains the main treatment. Although radiation can temporarily improve symptoms, tumors almost always return, and better ways to monitor and treat this disease are urgently needed. Extracellular vesicles (EVs) are small particles released by cells that carry biological information, including RNA and proteins. In DMG, EVs may help tumor cells communicate, adapt to radiation, and develop resistance. At the same time, EVs may provide a minimally invasive way to monitor tumor behavior through blood or cerebrospinal fluid and may eventually serve as delivery vehicles for new therapies. This review summarizes how EVs may contribute to DMG biology, radiation response, treatment resistance, and future approaches to diagnosis and therapy.

## 1. Introduction

DMG, including DIPG, represents one of the most aggressive and lethal primary brain tumors of childhood. Characterized by infiltrative growth within critical midline structures and frequently driven by histone H3K27 alterations [1,2,3], these tumors remain uniformly fatal despite decades of clinical investigation. Median survival remains less than 12 months, and current therapeutic strategies offer only transient benefit [4,5]. Radiotherapy continues to serve as the standard-of-care, providing temporary symptomatic and radiographic improvement, yet resistance inevitably develops, underscoring the urgent need for novel diagnostic and therapeutic approaches [6]. A major barrier to progress in DMG lies in both its anatomical inaccessibility and its biological complexity. Surgical resection is not feasible, and even biopsy has historically been limited, although it is increasingly performed in specialized centers [7,8]. As a result, there remains a critical unmet need for minimally invasive approaches to characterize tumor biology, monitor disease progression, and assess treatment response [1,7]. At the same time, the diffuse and infiltrative nature of DMG, coupled with the restrictive properties of the BBB, significantly limits the delivery and efficacy of systemic therapies [9,10].

EVs, including exosomes, have emerged as key mediators of intercellular communication in both physiological and pathological contexts [11,12]. These membrane-bound nanovesicles carry a diverse cargo of proteins, lipids, and nucleic acids reflective of their cell of origin and are capable of modulating recipient-cell behavior locally and at distant sites. In cancer, EVs have been implicated in processes central to tumor progression, including proliferation, invasion, immune modulation, and therapeutic resistance [13,14]. Importantly, EVs are detectable in biofluids such as blood and cerebrospinal fluid (CSF), positioning them as attractive candidates for liquid biopsy applications [15,16,17].

In the context of central nervous system malignancies, EVs are of particular interest due to their ability to traverse the BBB, enabling bidirectional communication between intracranial tumors and the systemic circulation [18,19,20]. This property provides a unique opportunity to access tumor-derived molecular information through minimally invasive approaches. In parallel, EVs have been increasingly explored as therapeutic delivery vehicles, owing to their intrinsic biocompatibility, low immunogenicity, and potential for targeted cargo delivery across biological barriers [21,22].

Emerging evidence suggests that EVs may actively modulate tumor responses to radiotherapy [23,24,25], the cornerstone of DMG treatment. Ionizing radiation has been shown to influence EV release and cargo composition [26], while EV-mediated signaling may contribute to adaptive resistance mechanisms, including enhanced DNA repair, metabolic reprogramming, and microenvironmental remodeling [27,28]. These findings raise the possibility that EVs not only reflect treatment response but may actively shape it [20,29].

**Figure 1:**
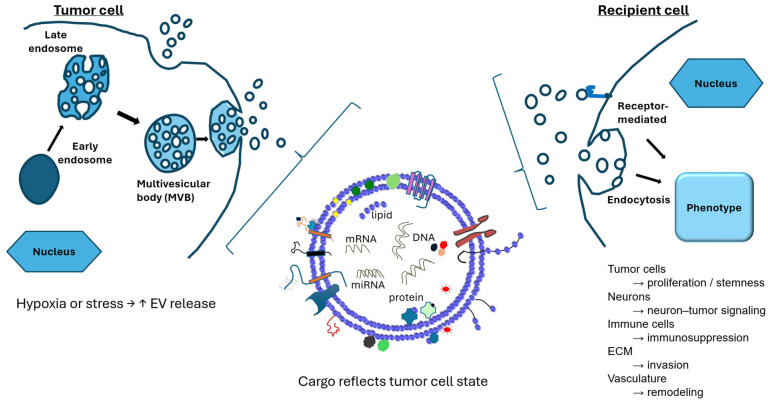
**Biogenesis, cargo, and functional roles of extracellular vesicles in diffuse midline glioma.** Extracellular vesicles (EVs), including exosomes, are generated through the endosomal pathway, in which early endosomes mature into multivesicular bodies (MVBs) that fuse with the plasma membrane to release vesicles. EV release may be enhanced under cellular stress, such as hypoxia. EVs carry diverse cargo—including miRNAs, mRNAs, proteins, lipids, and DNA—reflecting the molecular state of the parent tumor cell. Following release, EVs are taken up by recipient cells via mechanisms such as receptor-mediated endocytosis, enabling transfer of functional cargo. In diffuse midline glioma (DMG), EVs contribute to tumor proliferation and stemness, neuron–tumor signaling, immune suppression, extracellular matrix remodeling, and vascular adaptation, thereby coordinating tumor progression and microenvironmental interactions. Adapted from [33] under the terms of the Creative Commons Attribution License.

**Figure 2:**
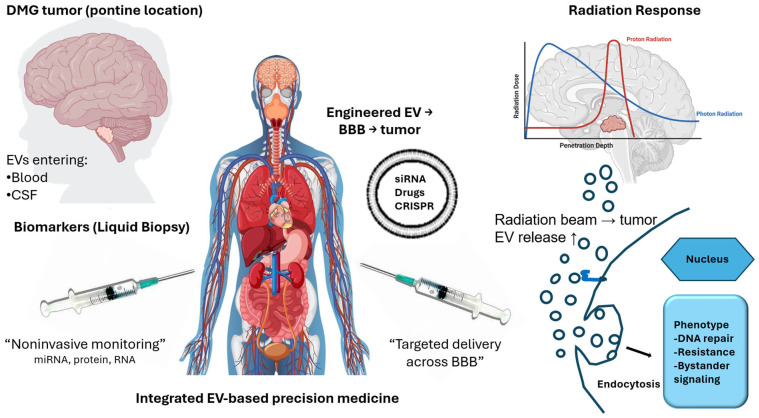
**Translational applications of extracellular vesicles in diffuse midline glioma.** Extracellular vesicles (EVs) play multiple roles across the translational spectrum of diffuse midline glioma (DMG). As biomarkers, tumor-derived EVs released into blood and cerebrospinal fluid (CSF) enable noninvasive monitoring through detection of RNA and protein cargo. As therapeutic delivery platforms, engineered EVs can transport small molecules (drugs), nucleic acids (e.g., siRNA), and gene-editing components across the blood–brain barrier to tumor cells. In the context of radiotherapy, EV release is increased following irradiation, and EV-mediated signaling contributes to adaptive responses, including enhanced DNA repair, treatment resistance, and bystander effects. Collectively, these functions position EVs as key components of integrated, EV-based precision medicine strategies in DMG. Adapted from [33] under the terms of the Creative Commons Attribution License.

In this review, we examine the role of EVs in DMG across the translational spectrum, from fundamental pathobiology to emerging clinical applications, including their potential as biomarkers, therapeutic delivery platforms, and modulators of radiation response. By integrating current preclinical and clinical evidence, we aim to highlight both the promise and the limitations of EV-based approaches in this challenging disease and to identify key directions for future investigation. A conceptual overview of EV biology and translational applications in DMG is provided in Figure 1 and Figure 2 [1,20].

## 2. Literature Search Strategy

A narrative literature review was conducted to evaluate the role of EVs in DMG, including DIPG. A comprehensive search of the PubMed and Scopus databases was performed to identify relevant articles published up to March 2026. Search terms included combinations of the following keywords: “diffuse midline glioma,” “DIPG,” “pontine glioma,” “extracellular vesicles,” “exosomes,” “liquid biopsy,” “biomarkers,” “radiotherapy,” and “radioresistance.” Titles and abstracts were screened for relevance, and full-text articles were reviewed when appropriate. Studies were included if they investigated EVs or exosomes in the context of DMG, DIPG, pediatric high-grade glioma, or related central nervous system tumors, with particular emphasis on tumor biology, biomarker development, therapeutic delivery, and radiation response. Due to the limited number of DMG-specific studies, selected literature from adult glioma and broader oncology contexts was also included to provide mechanistic and translational insight. In addition, because relatively few studies have directly examined extracellular vesicles in DMG, selected evidence from pediatric high-grade glioma, adult glioma, and other cancer models was included to provide mechanistic context. Throughout the manuscript, findings derived directly from DMG models are distinguished from those extrapolated from related tumor systems whenever possible. Reference lists of selected articles were manually reviewed to identify additional relevant publications. Only articles published in English were included. Preprints, conference abstracts, editorials, and non-peer-reviewed publications were excluded. Review articles were used primarily for background context and identification of relevant primary literature, but were not considered primary sources of evidence. Given the narrative nature of this review, formal meta-analysis and quantitative synthesis were not performed. As a narrative review, this manuscript is subject to potential selection bias, and studies were selected based on their relevance to EV biology, DMG, radiation response, therapeutic resistance, liquid biopsy, and translational applications. Lastly, many studies historically referred to these vesicles as exosomes; however, consistent with current International Society for Extracellular Vesicles (ISEV) recommendations, the term EVs is used throughout this review unless a specific vesicle subtype was experimentally defined.

### 2.1. EVs in the Pathobiology of DMG

EVs play a central role in mediating intercellular communication within the tumor microenvironment and have emerged as key contributors to the pathobiology of DMG [20]. Through the transfer of bioactive cargo, including microRNAs, long non-coding RNAs, proteins, and lipids, EVs enable tumor cells to influence neighboring malignant cells as well as non-neoplastic components of the central nervous system [30]. In DMG, where tumor growth is highly infiltrative and spatially heterogeneous, EV-mediated signaling likely plays a central role in coordinating tumor progression and adaptive responses (Figure 1) [12,31,32].

### 2.2. EVs and Tumor Cell Proliferation and Survival

EVs derived from cancer cells have been shown to promote tumor cell proliferation and survival through the transfer of oncogenic signaling molecules [13,16,34,35]. More specifically, EVs derived from glioblastoma cells have been shown to promote proliferation, stemness, mesenchymal transition, and therapeutic resistance through the transfer of proteins and regulatory RNAs capable of activating NF-κB/STAT3 and other oncogenic signaling pathways [36,37,38,39]. In DMG, characterized by epigenetic dysregulation associated with H3K27 alterations, EV-mediated transfer of regulatory molecules may further amplify aberrant transcriptional programs that support tumor growth [2,3]. For example, EV-associated microRNAs and long non-coding RNAs have been implicated in regulating chromatin remodeling, stemness, and proliferative signaling in glioma models, suggesting potential mechanisms by which EV cargo may influence epigenetically dysregulated DMG cells. In addition to direct effects on tumor cells, EVs may contribute to the maintenance of a stem-like tumor cell population, which is believed to play a role in therapeutic resistance and disease recurrence [39,40,41,42,43]. By modulating signaling pathways associated with self-renewal and differentiation, EVs may help sustain a population of cells with enhanced tumorigenic potential.

### 2.3. EV-Mediated Interactions with the Neural Microenvironment

A defining feature of DMG is its localization within the brainstem and its intimate interaction with the surrounding neural environment [8]. Recent studies have highlighted bidirectional communication between glioma cells and neurons [44], with neuronal activity influencing tumor growth [45]. EVs are increasingly recognized as mediators of this cross-talk. Tumor-derived EVs have been shown to directly influence central nervous system resident cells through the transfer of functional proteins and regulatory RNAs. In glioblastoma models, EVs released by tumor cells were taken up by microglia and macrophages in vivo, resulting in altered cytokine profiles, increased proliferation, and functional reprogramming of recipient cells, demonstrating a direct mechanism of tumor–microenvironment communication [46]. Subsequent studies further showed that EV-mediated transfer of miR-21 from glioma cells to microglia promotes microglial reprogramming through suppression of target genes associated with normal immune function, providing evidence that EV cargo can actively reshape the local neural microenvironment [47]. In addition to effects on microglia, glioblastoma-derived EVs have been shown to induce normal astrocytes to acquire a tumor-supportive phenotype through alterations in p53- and MYC-regulated signaling pathways, thereby promoting conditions favorable for tumor progression [48]. Collectively, these findings support a model in which EV-mediated communication between tumor cells and CNS-resident cells contributes to the establishment of a permissive neural microenvironment that facilitates glioma growth and survival.

### 2.4. EVs in Invasion and Diffuse Tumor Growth

Diffuse infiltration is a hallmark of DMG and a major obstacle to effective treatment [49]. EVs have been implicated in promoting invasive behavior through the transfer of molecules that regulate cytoskeletal dynamics, extracellular matrix remodeling, and cell motility [13]. By modulating the expression of adhesion molecules and proteolytic enzymes [50,51,52,53,54,55], EVs may facilitate tumor cell migration along white matter tracts and perivascular spaces. In addition, EV-mediated signaling can influence stromal and endothelial cells, contributing to the remodeling of the extracellular environment in a manner that favors tumor dissemination [51,53,54,55,56,57,58]. Experimental studies have demonstrated that EV-mediated transfer of proteins and regulatory RNAs can enhance migratory and invasive behavior in recipient glioma cells, supporting a role for EV signaling in the diffuse growth patterns characteristic of high-grade gliomas [50,56,57,58,59,60]. By transferring invasion-associated cargo, including integrins, CD44/HA-axis components, proteases, and cytoskeletal regulators, glioma-derived EVs can remodel the extracellular matrix and enhance tumor cell migration and invasion [51,52,53,54,55].

### 2.5. EVs and Immune Modulation in DMG

The tumor microenvironment of DMG is characterized by a relatively immunologically “cold” phenotype, with limited immune infiltration and an immunosuppressive milieu [61]. EVs may contribute to this environment by modulating the activity of resident and infiltrating immune cells. Tumor-derived EVs have been shown to suppress anti-tumor immune responses through multiple complementary mechanisms. Glioblastoma stem cell-derived EVs promote immunosuppressive polarization of monocytes and macrophages, inducing M2-like phenotypes, increasing PD-L1 expression, and activating STAT3 signaling pathways associated with immune evasion [62]. In parallel, EV-associated CD73 has been shown to inhibit T-cell clonal expansion and effector function through adenosine-mediated signaling, providing a direct mechanism by which glioma-derived EVs suppress adaptive immune responses [63]. Additional studies have demonstrated that EV-associated LGALS9 impairs dendritic cell antigen presentation and suppresses cytotoxic T-cell immunity, further limiting anti-tumor immune surveillance [64]. Collectively, these studies demonstrate that glioma-derived EVs suppress anti-tumor immunity through coordinated effects on monocytes, macrophages, dendritic cells, and T cells, thereby contributing to the establishment of an immunosuppressive tumor microenvironment.

### 2.6. EVs in Therapeutic Resistance

Beyond their roles in tumor growth and invasion, EVs are increasingly recognized as mediators of resistance to therapy [39,65]. By transferring resistance-associated molecules between cells, EVs may enable rapid adaptation to therapeutic stress [39,41,66]. In the context of DMG, EV-mediated mechanisms may contribute to resistance not only to systemic therapies but also to radiotherapy [20,43], as discussed in subsequent sections. The ability of EVs to disseminate resistance traits across tumor cell populations underscores their potential importance in disease progression and highlights them as potential therapeutic targets. A representative example is provided by H3K27M-mutant DMG models, in which small EVs derived from radioresistant cells transferred proteins, microRNAs, and metabolites associated with DNA repair and metabolic adaptation to radiosensitive recipient cells, thereby promoting resistance to radiation therapy [43]. Collectively, these findings underscore the central role of EVs in coordinating tumor growth, invasion, and microenvironmental adaptation in DMG (**Table 1**).

### 2.7. EVs as Biomarkers and Liquid Biopsy Tools in DMG

The development of reliable biomarkers for DMG remains a critical unmet need. Due to the eloquent anatomical location of these tumors, repeated tissue sampling is not feasible, limiting the ability to monitor disease progression, therapeutic response, and molecular evolution over time. In this context, liquid biopsy approaches have emerged as promising alternatives, with EVs representing a particularly attractive platform [11,18,20]. Because EV cargo reflects the molecular state of the parent tumor cell, EV-associated RNAs and proteins may provide information regarding tumor burden, molecular subtype, and treatment response that is not readily obtainable through conventional imaging alone [17,69,70].

### 2.8. Rationale for EV-Based Liquid Biopsy in DMG

EVs are released into the extracellular space by virtually all cell types, including tumor cells, and are detectable in a variety of biofluids, most notably plasma and CSF. Because EV cargo reflects the molecular composition of the parent cell, tumor-derived EVs may provide a real-time snapshot of tumor biology [11]. In DMG, EVs offer several theoretical advantages as biomarkers. Their lipid bilayer membrane protects nucleic acids and proteins from degradation, enabling more stable detection compared with free circulating molecules. Furthermore, EVs may traverse the BBB, allowing intracranial tumor signals to be detected in peripheral blood [18,19,22]. These properties position EVs as candidates for minimally invasive, longitudinal disease monitoring. These advantages are particularly relevant in DMG, where repeated tissue sampling is generally not feasible and serial assessment of tumor evolution remains a major clinical challenge [18,19,22].

### 2.9. EV Cargo as a Source of Diagnostic and Prognostic Information

EVs contain a diverse array of biomolecules, including microRNAs, long non-coding RNAs, messenger RNAs, proteins, and lipids. In glioma and other central nervous system tumors, EV-associated microRNAs have been among the most extensively studied analytes, with several candidates proposed as potential diagnostic or prognostic markers [17,69,70]. In the context of DMG, emerging studies suggest that EV-associated RNA and protein signatures may correlate with tumor burden, molecular subtype, and disease progression. However, compared with adult glioblastoma and other malignancies [20], the DMG-specific EV biomarker literature remains relatively limited. Nevertheless, studies in glioma have identified EV-associated microRNAs, proteins, and other molecular signatures that correlate with disease progression and clinical outcome, supporting the potential utility of EV cargo as a source of diagnostic and prognostic information in DMG [17,20,69,70]. As such, many current insights are extrapolated from broader glioma or pediatric high-grade glioma studies.

### 2.10. CSF Versus Plasma EVs

Both CSF and plasma represent viable sources of EVs for biomarker development, each with distinct advantages and limitations. CSF is in closer proximity to the tumor and may therefore provide a higher concentration of tumor-derived EVs, potentially enhancing sensitivity [71]. However, CSF collection is invasive and not always feasible for repeated sampling. In contrast, plasma-based EVs analysis offers a more practical and minimally invasive approach but may be limited by dilution with EVs derived from non-tumor sources. The relative performance of CSF versus plasma EVs in DMG remains an area of active investigation [72], and future studies will be needed to determine the optimal sampling strategy.

### 2.11. Comparison with Circulating Tumor DNA (ctDNA)

ctDNA, particularly when derived from CSF, has emerged as a promising biomarker modality in DMG, enabling detection of characteristic mutations such as H3K27 alterations [72,73]. Compared with ctDNA, EV-based biomarkers offer complementary advantages, including the ability to capture functional and regulatory molecules, such as RNA and proteins, rather than genomic alterations alone [74]. For example, EV-associated microRNAs, proteins, and RNA signatures have been reported to correlate with tumor burden, molecular subtype, and disease progression in glioma, providing biologic and functional information that may complement the mutation-based profiling obtained through ctDNA analysis [17,20,69,70]. However, ctDNA assays are currently more advanced in terms of clinical validation in DMG [73]. EV-based approaches, while promising, face challenges related to sensitivity, specificity, and standardization. A combined approach integrating EV-derived analytes with ctDNA and other biomarker modalities may ultimately provide the most comprehensive assessment of disease status.

### 2.12. Current Limitations and Future Directions

Despite their potential, several barriers currently limit the clinical implementation of EV-based biomarkers in DMG. Key limitations include the lack of standardized methods for EV isolation and characterization, variability in EV yield and composition across biofluids and patients, limited availability of DMG-specific validation cohorts [20], and challenges in distinguishing tumor-derived EVs from background vesicles [12,15,18]. Future efforts should focus on the development of robust, reproducible workflows, as well as the identification of tumor-specific EV signatures. Integration with multi-omic approaches and longitudinal sampling strategies will likely be essential for realizing the full clinical potential of EV-based liquid biopsy in DMG. Representative EV-associated biomarkers relevant to DMG and related gliomas are summarized in **Table 2**.

### 2.13. EVs as Therapeutic Delivery Platforms in DMG

Therapeutic progress in DMG remains severely constrained by the inability to effectively deliver agents to tumor cells within the central nervous system. The BBB restricts the penetration of most systemic therapies, while the diffuse and infiltrative nature of these tumors further complicates targeted delivery. In this context, EVs have emerged as promising, biocompatible delivery systems capable of overcoming key barriers to effective therapy [18,29].

### 2.14. The BBB and Therapeutic Limitations in DMG

The BBB is a highly selective physiological barrier that protects the central nervous system but also significantly limits the entry of therapeutic agents, including many chemotherapeutics and biologics [76]. Although some degree of BBB disruption may occur in high-grade gliomas, DMG tumors often retain regions of intact barrier function, leading to heterogeneous drug distribution [19,20,21,22]. Traditional strategies to overcome the BBB, such as high-dose systemic therapy, convection-enhanced delivery, or focused ultrasound, have shown limited success or present substantial technical challenges [77]. As a result, there is a critical need for delivery platforms that can efficiently traverse the BBB and selectively target tumor cells.

### 2.15. Advantages of EV-Based Delivery Systems

EVs possess several intrinsic properties that make them highly attractive as therapeutic delivery vehicles in DMG. As naturally occurring membrane-bound vesicles, EVs exhibit excellent biocompatibility and low immunogenicity, reducing the likelihood of adverse immune responses compared with synthetic nanoparticle systems [16]. This feature is particularly advantageous for repeated or systemic administration. A defining characteristic of EVs is their ability to traverse the BBB, a major obstacle in the treatment of central nervous system malignancies [21,29]. Evidence from experimental delivery models indicates that some engineered or targeted EV preparations can cross the BBB or accumulate within the central nervous system, although the precise transport mechanisms, including the contribution of transcytosis, remain incompletely defined and may vary by EV source, surface modification, and disease context [21], enabling the delivery of therapeutic cargo to intracranial tumor sites that are otherwise difficult to access. In addition, the lipid bilayer structure of EVs provides protection for encapsulated cargo, including RNA, proteins, and small-molecule drugs, shielding these molecules from enzymatic degradation and improving their stability in circulation [78,79]. This protective capacity enhances the likelihood of successful delivery to target cells. EVs also offer potential for targeting specificity [18,19,20,21,22,80,81,82,83]. Surface proteins and membrane components can be engineered or selectively enriched to promote preferential uptake by tumor cells or other cells within the tumor microenvironment. Such targeting strategies may improve therapeutic efficacy while minimizing off-target effects. Collectively, these properties position EVs as a versatile and promising platform for therapeutic delivery in DMG, with the potential to overcome key biological barriers that have historically limited treatment efficacy [18,19,22].

### 2.16. Preclinical EV-Based Therapeutic Strategies in DMG

A growing body of preclinical research has explored the use of EVs as delivery vehicles in DMG and related pediatric high-grade gliomas. One notable strategy involves the use of macrophage-derived EVs engineered to carry both small-molecule inhibitors and nucleic acid therapeutics. For example, Shan et al. demonstrated that functionalized macrophage-derived exosomes loaded with panobinostat and PPM1D-siRNA improved delivery to DIPG models and enhanced anti-tumor efficacy compared with free drug approaches [84]. In addition to small-molecule drugs, EVs have been used to deliver siRNA [66,85], microRNA mimics or inhibitors targeting oncogenic pathways [27,28], CRISPR/Cas components for early-stage gene-editing applications [86], and protein therapeutics including enzymes or signaling modulators [87,88]. Collectively, these approaches highlight the flexibility of EVs as carriers capable of delivering diverse therapeutic payloads.

### 2.17. Engineering and Targeting of EVs

To enhance therapeutic efficacy, EVs can be engineered to improve cargo loading, stability, and targeting specificity [80]. Cargo loading strategies include both endogenous approaches [81,83], where donor cells are genetically modified to produce EVs containing desired cargo, and exogenous approaches, such as electroporation or chemical loading of isolated vesicles [82]. Targeting strategies often involve the modification of EV surface proteins to promote selective uptake by tumor cells. For example, incorporation of targeting ligands or peptides that recognize tumor-associated receptors may enhance delivery efficiency and reduce off-target effects [43,74]. In the context of DMG, where tumor cells are dispersed throughout critical brain structures, achieving effective targeting remains a significant but essential challenge.

### 2.18. Challenges and Translational Barriers

Despite their promise, several challenges must be addressed before EV-based therapies can be translated into clinical practice, including scalability and manufacturing, heterogeneity of EV populations, cargo loading efficiency, biodistribution and off-target effects, and evolving regulatory considerations [12,15]. Addressing these challenges will be critical for advancing EV-based delivery systems from preclinical models to clinical application in DMG.

### 2.19. Future Perspectives: Integrating EVs with Multimodal Therapy

The future of EV-based therapeutic delivery in DMG is likely to involve integration with existing treatment modalities, particularly radiotherapy. EVs could be used to deliver radiosensitizing agents [29,43], modulate resistance pathways, or enhance immune responses in combination with radiation [23,25]. In addition, personalized EV-based therapies tailored to the molecular profile of individual tumors may represent a step toward precision medicine in this disease [15,89]. As our understanding of EV biology continues to evolve, these vesicles hold significant promise as a platform for overcoming some of the most formidable challenges in DMG therapy. Key EV-based therapeutic delivery strategies are summarized in Table 3.

### 2.20. EVs and Radiation Response in DMG

Radiotherapy remains the cornerstone of treatment for DMG, providing transient clinical and radiographic improvement in the majority of patients. However, the near-universal recurrence of disease following irradiation reflects the rapid emergence of radioresistance, a defining feature of DMG biology. Given the limited availability of DMG-specific datasets, many mechanistic insights are extrapolated from glioblastoma and other tumor models. Accumulating evidence indicates that EVs contribute to both adaptive radiation responses and the propagation of radioresistant phenotypes within the tumor microenvironment.

### 2.21. Radiation-Induced EV Release and Radioresistance Transfer

Ionizing radiation induces cellular stress primarily through DNA damage, including direct double-strand breaks and indirect effects mediated by reactive oxygen species generated during water radiolysis [90,91]. Beyond these direct cytotoxic effects, radiation also initiates intercellular signaling processes that extend to neighboring, non-irradiated cells, a phenomenon known as the radiation-induced bystander effect. Increasing evidence suggests that EVs are key mediators of this communication [91,92,93]. In addition, the ability to overcome radioresistance through dose escalation is limited in DMG, as normal brainstem radiation tolerances constrain treatment intensity due to the risk of radiation-induced necrosis.

Radiation has been shown to alter both the quantity and composition of EVs released by tumor cells. In glioma and other cancer models, irradiation can increase EV secretion, although the magnitude and timing of this response appear to depend on factors such as radiation dose, cellular context, and post-irradiation interval [94,95,96]. In parallel, radiation-induced EVs exhibit distinct cargo profiles, including changes in microRNAs, proteins involved in DNA damage response pathways, and metabolic regulators. Notably, EVs derived from irradiated cells may also demonstrate enhanced uptake by recipient cells, suggesting increased efficiency of intercellular transfer following radiation exposure [94,97].

These alterations in EV release and cargo composition provide a mechanism by which irradiated tumor cells can transmit stress-adaptive signals to surrounding cells. Through the transfer of bioactive molecules, EVs may propagate radiation-induced signaling within the tumor microenvironment, contributing to bystander effects and influencing cellular responses beyond the directly irradiated population [98,99,100]. Collectively, these findings support a model in which radiation not only impacts tumor cells directly but also reshapes intercellular communication networks through EV-mediated signaling.

## 3. Radiation-Induced Modulation of EV Cargo

Although direct studies in DMG remain limited, accumulating evidence from multiple cancer models demonstrates that ionizing radiation can induce selective and functionally relevant changes in EV cargo. In several tumor types, including head and neck cancer [101] and neuroblastoma [102], EVs derived from irradiated cells exhibit altered protein and microRNA profiles, with enrichment of molecules involved in DNA repair, oxidative stress responses, chromatin organization, and cellular metabolism [103,104,105]. These radiation-induced changes in EV composition suggest a role for EVs in propagating stress-adaptive signaling within the tumor microenvironment. Thus, radiation-induced EV signaling may be viewed as part of a broader adaptive stress-response network that includes DNA damage signaling, oxidative stress adaptation, metabolic rewiring, immune modulation, and intercellular transfer of resistance-associated phenotypes [43,91,98,99,100,103,104,105,106]. A recent review further highlighted the role of radiation-induced extracellular vesicle signaling in mediating cellular stress responses, intercellular communication, and treatment adaptation across multiple tumor types [106].

Importantly, emerging studies in pediatric DMG models, particularly those harboring H3K27 alterations, provide more direct evidence that small EVs can mediate the transfer of radioresistant phenotypes [43]. EVs derived from radioresistant DMG cells have been shown to enhance survival and reduce radiation sensitivity in recipient cells, implicating the transfer of bioactive cargo capable of modulating DNA damage response pathways, redox homeostasis, and metabolic adaptation [43].

Together, these findings support a model in which EVs function as vectors for the horizontal transfer of resistance traits, amplifying the influence of resistant subclones within a heterogeneous tumor population. In the context of DMG, where diffuse infiltration and cellular heterogeneity are defining features [1,4], EV-mediated communication may play a particularly important role in shaping treatment response and promoting the emergence of radioresistance.

### 3.1. EVs and Microenvironmental Contributions to Radiation Response

Beyond tumor cell-intrinsic effects, EVs may influence radiation response through interactions with the tumor microenvironment [46,47,69,107]. EV-mediated signaling can modulate the behavior of surrounding cells, including astrocytes, microglia, and infiltrating immune populations [61,62,63,64,108,109,110], thereby shaping a microenvironment that supports tumor survival following irradiation. In particular, EVs may promote the establishment of a pro-survival and immunosuppressive niche [43,111,112,113], attenuating anti-tumor immune responses and facilitating tumor recovery after radiation-induced injury.

Emerging evidence suggests that EVs derived from cells within the tumor microenvironment can directly influence tumor cell radioresponse. For example, EVs released by tumor-associated macrophages, particularly M2-polarized populations, have been shown to enhance tumor cell survival and promote radioresistance through activation of pro-survival signaling pathways [75,114,115]. In parallel, though not yet described in DMG or DIPG, radiation-induced EVs can modulate immune signaling by influencing key regulators of T-cell activation and co-stimulation, including CD28, CD86, CD47, OX40L, 4-1BBL, and GITRL, thereby affecting immune activation, persistence, and evasion [116,117,118,119].

Collectively, these findings highlight a complex and context-dependent role for EVs in mediating microenvironmental responses to radiation. While EV-mediated signaling may contribute to antitumor immune activation under certain conditions, it may also reinforce immunosuppressive pathways that promote tumor persistence and radioresistance [113]. In addition, EVs may facilitate vascular and metabolic adaptations that enable tumor cells to withstand radiation-induced stress, further underscoring their role in shaping treatment outcomes in DMG.

### 3.2. EVs as Biomarkers of Radiation Response

Reliable biomarkers for monitoring radiation response in DMG remain limited. Imaging typically detects changes at relatively late time points, and repeated biopsies are not feasible due to the tumor’s anatomical location. Liquid biopsy approaches offer a minimally invasive alternative, and EVs may provide real-time insight into tumor radiation response.

Tumor-derived EVs are detectable in blood and CSF and carry molecular cargo that reflects the functional state of tumor cells [120]. Unlike circulating nucleic acids, which primarily indicate tumor burden, EVs may capture active biological processes, including signaling pathway activity, metabolic states, and cellular stress responses [43]. These properties position EVs as a potentially dynamic biomarker platform capable of monitoring radiation response over time, identifying emerging resistance mechanisms, and informing adaptive therapeutic strategies.

Emerging evidence further suggests that EVs may function not only as biomarkers but also as mediators of radiation response. Small EVs derived from radioresistant H3K27M-mutant DMG cells have been shown to transfer resistance-associated phenotypes to radiosensitive cells through the delivery of proteins, microRNAs, and metabolites involved in DNA repair and metabolic pathways such as oxidative phosphorylation and glycolysis [43]. Upon uptake, these vesicles may reprogram recipient cells and enhance survival following radiation, indicating that EV cargo reflects molecular programs associated with radiation resistance. In addition, radiation has been reported to increase EV release and uptake, potentially enhancing the transfer of resistance-associated signals between tumor cells and contributing to adaptive tumor survival [43,67,68].

In the randomized phase 2 BIOMEDE trial, patients with biopsy-proven DIPG were stratified based on molecular biomarkers and treated with targeted therapies, including EGFR inhibitors, mTOR inhibitors, and multi-kinase inhibitors, in combination with radiotherapy [121]. This study highlights the growing importance of biomarker-driven treatment strategies and underscores that radiotherapy not only functions as a cytotoxic modality but may also influence tumor signaling pathways and the tumor microenvironment. Although no overall survival benefit was observed across treatment arms, the BIOMEDE trial identified TP53 mutation as a strong adverse prognostic biomarker. The study further suggested that patients with elevated PI3K/AKT/mTOR pathway activity who received mTOR inhibition exhibited improved progression-free and overall survival, supporting the potential of this pathway as a predictive theranostic biomarker [121].

Complementing these findings, H3K27M DMG tumors exhibit marked cellular heterogeneity, with stem-like oligodendrocyte precursor cell (OPC)-like populations associated with increased tumor aggressiveness and poorer survival outcomes [122]. These tumorigenic states are enriched for signaling pathways such as PI3K/mTOR, MYC, DNA repair, and cell cycle regulation, as well as metabolic programs including oxidative phosphorylation and cholesterol biosynthesis [122].

Collectively, these observations suggest that PI3K/AKT/mTOR pathway activation represents a biologically relevant and potentially targetable state in DMG, although the mechanisms regulating this pathway within the tumor microenvironment remain incompletely defined. Tumor-derived EVs have been shown to contain PI3K/AKT/mTOR pathway transcripts that reflect tumor gene expression, supporting their role in intercellular signaling and their potential utility as noninvasive biomarkers [123,124]. In glioma models, EVs have been reported to promote proliferation and migration of recipient cells through activation of the PI3K/AKT/mTOR pathway [123]. Although EVs are associated with PI3K/AKT/mTOR pathway activity and may induce signaling in recipient cells, the specific cargo responsible for these effects remains poorly defined. Further characterization of EV-associated proteins, RNAs, and lipids that drive pathway activation will be important for the development of EV-based biomarkers and targeted therapies.

Radiotherapy modulates multiple molecular pathways in tumor cells. Ionizing radiation induces DNA damage responses that activate ATM/ATR signaling and downstream JAK/STAT pathways, while also engaging interferon signaling, cGAS–STING activation, and EGFR-mediated PI3K/AKT/mTOR signaling [125]. Radiation has also been shown to increase EV release and alter EV cargo composition, and EV-mediated signaling may influence key survival pathways, including PI3K/AKT/mTOR; however, whether radiation-induced EVs directly drive these signaling changes remains incompletely defined [91]. Radiation may alter the composition and function of tumor-derived EVs, thereby influencing intercellular communication within the tumor microenvironment. Given the limited feasibility of repeated tumor biopsies in DMG, EVs represent a promising minimally invasive approach to monitor dynamic changes in tumor signaling during treatment. As such, EVs may serve as real-time biomarkers of radiation response and represent potential targets for limiting radiation-driven tumor adaptation. In future studies, EV-based biomarkers may also help distinguish tumor recurrence from treatment-related imaging changes such as pseudoprogression, although this application remains largely unexplored in DMG.

### 3.3. Radiation-Induced Immune Modulation and EV-Mediated Resistance in DMG

A defining feature of the DMG immune microenvironment is the relative paucity of lymphocytes, as studies have demonstrated low T-cell infiltration in both pretreatment biopsies and postmortem tumor samples [115]. Despite robust major histocompatibility complex (MHC) class I expression in DIPG tumors, CD8-positive T cells remain infrequent compared with adult glioblastoma, suggesting that antigen presentation alone is insufficient to generate an effective endogenous antitumor T-cell response [115]. This lymphocyte-poor state may partly explain why PD-1 blockade has demonstrated limited activity in DMG, as checkpoint inhibition typically requires a pre-existing or inducible antitumor immune response [126]. In addition, DIPG may impair T-cell function at the immune synapse level, as chimeric antigen receptor (CAR) T-cell studies have reported reduced calcium flux, impaired lysosome recruitment, and diminished effector activation against DIPG cells [115].

Radiation may partially overcome some of these immune barriers by increasing tumor antigen release, enhancing T-cell activation, and promoting immune cell infiltration into the tumor microenvironment. Radiotherapy has been shown to modulate immune responses by enhancing T-cell activation, localization, and function within tumors [127]. Mechanistically, radiation can induce immunogenic cell death, increase antigen availability, promote the release of danger-associated molecular patterns (DAMPs), and activate cGAS–STING-mediated type I interferon signaling, thereby supporting dendritic cell activation and T-cell priming [127,128]. Radiation may also facilitate T-cell trafficking by altering tumor vasculature and increasing chemokine signals that support immune cell recruitment [127]. In brain tumors, these effects may collectively enhance antitumor immunity by increasing tumor antigen exposure, promoting DAMP release and interferon signaling, and enabling activated T cells to access the central nervous system through a disrupted BBB [128].

In addition, clinically relevant X-ray doses have been shown to induce store-operated calcium entry in human T cells, a process in which depletion of intracellular calcium stores triggers calcium influx from the extracellular space. This signaling event promotes nuclear translocation of nuclear factor of activated T cells (NFAT), a key regulator of immune-related gene expression [129]. However, radiotherapy may also exert immunosuppressive effects by recruiting suppressive myeloid populations, expanding regulatory T cells, and inducing lymphopenia [127]. As such, although radiation may help convert an immunologically “cold” tumor microenvironment into a more permissive state, these effects may be insufficient to overcome the profound lymphocyte scarcity and immunosuppression observed in DMG. The net impact of radiotherapy on immune activation is therefore likely to depend on factors such as radiation dose, fractionation, timing, and combination with immunotherapeutic strategies.

In contrast to its limited lymphocyte infiltration, DMG is enriched in myeloid cell populations. CD11b-positive myeloid cells constitute a larger proportion of tumor-associated leukocytes in DIPG compared with adult glioblastoma, whereas T-cell and natural killer (NK) cell infiltration remains limited [115]. Microglia and macrophages accumulate within the DIPG tumor microenvironment but do not conform strictly to classical M1 (pro-inflammatory) or M2 (anti-inflammatory) polarization states [115]. Instead, these cells exhibit heterogeneous functional phenotypes, including disease-associated and proliferative states that may support tumor progression and immune suppression [115]. Reduced IL-2 expression may impair T-cell recruitment or maintenance, while DIPG-associated macrophages secrete lower levels of inflammatory mediators compared with macrophages in adult high-grade glioma [115].

This myeloid-dominant immune landscape provides important context for radiation-induced EV signaling. While radiation may enhance antigen availability and promote immune activation, tumor-derived EVs released following irradiation may also carry immunomodulatory signals that attenuate antitumor immunity. In breast cancer models, microparticles derived from irradiated tumor cells exhibited increased levels of immunomodulatory proteins, including PD-L1, and suppressed cytotoxic T-cell activation and granzyme B production [130]. In glioblastoma models, radiation increased the expression of immune-suppressive markers such as PD-L1 and CD47 on tumor cells, and tumor-derived EVs were preferentially taken up by myeloid cells within the tumor microenvironment [75]. Macrophages exposed to glioblastoma-derived EVs demonstrated altered cytokine secretion, increased phagocytic activity, and elevated PD-L1 expression, consistent with a shift toward an immunosuppressive phenotype [75].

Taken together, these findings suggest that radiation-induced EVs may represent a context-dependent mechanism in DMG. While radiation may enhance immune recognition through increased antigen exposure, EVs released from irradiated tumor cells may concurrently suppress cytotoxic T-cell activity and promote immunosuppressive reprogramming of macrophages and microglia. Although many of these observations are derived from breast cancer and glioblastoma models, they are likely relevant to DIPG given its lymphocyte-poor, low-inflammatory, and myeloid-dominant microenvironment. These findings support the possibility that EV-mediated signaling contributes to immune resistance in DIPG and provide a rationale for combining radiotherapy with strategies targeting EV-mediated immunosuppression, myeloid reprogramming, or immune checkpoint pathways.

### 3.4. Therapeutic Implications: Targeting EV-Mediated Radioresistance

Targeting EV-mediated signaling may represent a strategy to limit radiation-induced tumor adaptation in DMG. Small EVs derived from radioresistant H3K27M-mutant DMG cells have been shown to transfer resistance-associated proteins, microRNAs, and metabolites to radiosensitive cells, supporting the concept that radioresistance may be propagated through intercellular communication rather than arising solely from cell-intrinsic mechanisms [43]. Because radiation can increase EV release and uptake, this process may be further amplified following treatment and contribute to adaptive tumor survival [43,67,68].

Therapeutically, EV-mediated radioresistance could be targeted at multiple levels. Inhibition of EV production, release, or uptake may reduce the transfer of resistance-associated cargo between tumor cells and components of the tumor microenvironment [43,75,91]. Targeting EV-associated signaling pathways may also be important, particularly those involved in PI3K/AKT/mTOR signaling, DNA repair, oxidative phosphorylation, glycolysis, and immune checkpoint regulation [43,75,123]. This is clinically relevant given findings from the BIOMEDE trial, which suggest that PI3K/AKT/mTOR pathway activity may identify a subset of DIPG patients who derive benefit from mTOR inhibition [121].

EV-mediated immune suppression further supports the rationale for combining radiotherapy with immunomodulatory approaches. While radiation may enhance antigen availability and support immune activation, radiation-induced EVs may counteract these effects by suppressing cytotoxic T-cell activity or promoting immunosuppressive reprogramming of macrophages and microglia [75,130]. As such, therapeutic strategies targeting PD-1/PD-L1 signaling, CD47-mediated phagocytosis resistance, myeloid cell polarization, or EV-mediated communication may help preserve the immunostimulatory effects of radiation while limiting compensatory immune resistance [75,130].

Overall, EVs are increasingly recognized as active mediators of radiation adaptation rather than passive biomarkers. Future studies should aim to define the EV populations and cargo that contribute to radioresistance in DMG, determine whether these signatures can be reliably detected in blood or CSF, and evaluate whether targeting EV-mediated signaling enhances radiotherapy response. Given the limited feasibility of repeated tumor biopsies in DMG, EVs may provide a clinically useful platform for monitoring treatment response and guiding rational combination therapies aimed at reducing tumor survival, immune escape, and disease recurrence [43,120].

## 4. Current Limitations and Translational Challenges

Despite the growing interest in EVs as diagnostic and therapeutic tools in DMG, several significant challenges currently limit their clinical translation. One of the primary obstacles is the heterogeneity of EV populations [12,31,32]. EVs vary in size, cellular origin, and molecular composition, complicating their isolation, characterization, and functional interpretation [12,15,69,71,72,131,132]. Distinguishing tumor-derived EVs from those released by normal cells remains particularly challenging in biofluids such as plasma, where background vesicle populations are abundant [66,131,133,134,135,136,137]. This issue may be especially relevant in DMG, where tumor burden is relatively small, BBB integrity may remain partially preserved, and circulating tumor-derived EV abundance may be low. Strategies such as enrichment for tumor-associated surface markers, analysis of mutation-associated or tumor-enriched cargo, and integration of EV profiling with ctDNA and proteomic biomarkers may improve specificity and sensitivity in future liquid biopsy studies.

A related limitation is the lack of standardized methodologies for EV isolation and analysis [12,15]. Techniques such as ultracentrifugation, size-exclusion chromatography, and immunoaffinity capture yield EV populations with differing purity and composition, making cross-study comparisons difficult. These methodological differences can influence downstream RNA, protein, and lipid readouts, particularly in low-input biofluids such as CSF, and underscore the need for reproducible quality-control metrics and standardized reporting before EV-based biomarkers can be implemented clinically [12,15,131]. The absence of universally accepted protocols and biomarkers for EV characterization further contributes to variability in reported findings.

In the context of DMG specifically, a major limitation is the relative scarcity of disease-specific EV studies [20]. Much of the current understanding is extrapolated from adult glioblastoma or broader glioma research [69,137]. While these studies provide valuable mechanistic insights, DMG possesses distinct biological features, including H3K27 alterations and unique microenvironmental interactions [4,61], which may influence EV biology in ways not fully captured by existing models.

For biomarker applications, challenges include limited sensitivity and specificity, as well as the need for robust validation in large, well-characterized patient cohorts [69,71,72,132]. Although CSF may provide a more enriched source of tumor-derived EVs, its collection is invasive and may not be feasible for routine monitoring [135]. Plasma-based assays, while more practical, face issues related to dilution and background noise [135,136,137].

In therapeutic applications, significant barriers remain in scalable EV production, cargo loading efficiency, and targeting specificity. Ensuring consistent manufacturing of EV-based therapeutics with defined composition and function is essential for clinical translation. In addition, concerns regarding biodistribution, off-target effects, and long-term safety must be addressed [81,134].

Finally, regulatory considerations represent an additional challenge, as EV-based therapies occupy a complex space between biologics and drug delivery systems [133,138,139]. Clear frameworks for evaluation and approval are still evolving. Overcoming these limitations will require coordinated efforts to standardize methodologies, validate findings in DMG-specific contexts, and develop scalable, clinically applicable EV-based platforms [138,140]. Continued advances in EV biology and technology will be critical for translating these approaches into clinically relevant strategies for DMG. The diverse translational applications of EVs in DMG are summarized in **Figure 2**.

## 5. Conclusions

DMG remains one of the most challenging malignancies in neuro-oncology, with limited therapeutic options and poor clinical outcomes. EVs have emerged as important regulators of tumor biology and provide a compelling link between mechanistic insight and clinical application. Across the translational spectrum, EVs contribute to tumor growth, invasion, and microenvironmental remodeling, while also offering opportunities for minimally invasive biomarker development, targeted therapeutic delivery, and improved understanding of radiation response. Their ability to cross the BBB and reflect tumor-associated molecular changes makes them particularly attractive in DMG, where tissue access is limited and treatment options remain inadequate. At the same time, the field remains constrained by challenges related to standardization, specificity, scalability, and clinical validation. Addressing these issues will be essential before EV-based approaches can be integrated reliably into diagnostic or therapeutic workflows. Several priorities should guide future investigation. Standardization of EV isolation, characterization, and reporting methodologies will be essential to improve reproducibility across studies. Equally important is the establishment of larger DMG-specific validation cohorts and longitudinal plasma- and CSF-based studies to define clinically meaningful EV signatures associated with disease progression and treatment response. Finally, integration of EV-derived biomarkers with complementary liquid biopsy approaches, including ctDNA analysis, may provide a more comprehensive framework for monitoring tumor evolution and guiding precision therapeutic strategies. Emerging approaches, including single-cell and spatial profiling, multi-omic EV analysis, and computational integration of EV-derived biomarkers with genomic and proteomic data, may further refine future precision-monitoring strategies in DMG. Addressing these challenges will be critical for translating EV-based approaches into clinically actionable tools for patients with DMG. Even so, the rapid expansion of EV research suggests that these vesicles may ultimately become an important component of future DMG management. As the field advances, EV-based diagnostics and therapeutics may help reshape how this devastating disease is detected, monitored, and treated.

## Figures and Tables

**Table 1:** **Proposed roles of extracellular vesicles in the pathobiology of** **DMG.**

Category	EV Cargo/Mechanism	Biological Effect	Model/System	Key Implication	Reference
**Tumor proliferation**	miRNAs, oncogenic proteins	Enhanced cell growth and survival	Glioblastoma Multiforme (GBM)	EVs amplify oncogenic signaling	[11,37,38,42]
**Stemness**	Regulatory RNAs	Maintenance of stem-like tumor cells	GBM, Glioma stem cell models	Supports recurrence and resistance	[37,38,39,42]
**Neural interaction**	EV-mediated neuron–tumor signaling	Increased tumor proliferation	CNS tumor models, DMG/Glioma, GBM	Neural activity promotes tumor growth	[**44**,^46^,^47^,^48^]
**Invasion**	Proteases, adhesion molecules	Increased migration and infiltration	Glioma, GBM	Facilitates diffuse growth pattern	[50,51,52,53,54,55]
**Microenvironment remodeling**	Cytokines, signaling molecules	Stromal and endothelial activation	GBM and Glioma Tumor microenvironment models	Creates permissive niche	[46,47,48,53,56,57,58]
**Immune modulation**	Immunosuppressive proteins/miRNAs	Reduced immune activation	Glioma immune models	Promotes immune evasion	[**61**,^62^,^63^,^64^]
**Therapy resistance**	Stress-response molecules, DNA repair factors	Resistance to therapy	DMG/glioma models	EVs propagate resistance phenotypes	[39,40,41,42,**43**]
**Radiation response**	Altered EV cargo post-irradiation	Transfer of radioresistance	H3K27M DMG models	EVs contribute to treatment failure	[**43**,**67**,**68**]

**Bolded** references indicate studies performed directly in DMG/DIPG or H3K27-altered diffuse midline glioma models. All other references represent supporting evidence from glioblastoma, adult glioma, or broader extracellular vesicle literature.

**Table 2:** **Extracellular vesicle-associated biomarkers in DMG and related** **gliomas.**

Biomarker Type	EV Cargo	Biofluid	Proposed Clinical Use	Key Findings	Limitations	Reference
**microRNA**	miR-21, miR-124, miR-222 (glioma-associated)	Plasma	Diagnostic/disease monitoring	Differential expression in glioma patients vs controls; reflects tumor biology	Limited DMG-specific validation	[17,69,70]
**microRNA**	miR-21, miR-222	CSF	Tumor burden/progression	Enriched in tumor-derived EVs in CNS malignancies	Small cohorts; lack of standardization	[70,72,73]
**mRNA**	EGFRvIII transcripts (glioblastoma models)	Plasma	Molecular profiling	Detectable in EVs; reflects tumor genotype	Rare in DMG; extrapolated data	[11,69,70]
**Protein**	PD-L1	Plasma	Immune profiling	EV-associated PD-L1 correlates with immune suppression	Limited DMG-specific data	[62,63,64,75]
**Protein**	Tumor-associated proteins (e.g., GFAP, others)	CSF	Diagnostic adjunct	EV proteins reflect tumor origin	Specificity concerns	[69,70,71]
**Lipids**	Phospholipid signatures	Plasma	Exploratory biomarker	Altered lipid composition in tumor EVs	Early-stage research	[69,70,74]
**Multi-omic EV profiling**	RNA + protein panels	CSF/plasma	Integrated liquid biopsy	Potential for longitudinal monitoring	Lack of validated panels	

**Table 3:** **Extracellular vesicle-based therapeutic delivery strategies in DMG and related glioma** **models.**

Strategy	EV Source	Therapeutic Cargo	Target/ Mechanism	Model System	Key Findings	Translational Limitations	Reference
**Macrophage-derived EVs**	Macrophages	Panobinostat + PPM1D siRNA	Epigenetic modulation + DNA damage pathway inhibition	DMG/DIPG	EVs crossed BBB, delivered cargo, inhibited tumor growth	Scalability; targeting specificity	[84]
**Tumor cell–derived EVs (engineered)**	Glioma cells	siRNA/miRNA	Gene silencing of oncogenic pathways	Glioma models	Efficient cellular uptake and gene knockdown	Risk of tumor-promoting signals	[66,85]
**Mesenchymal stem cell EVs**	MSCs	Anti-tumor miRNAs/drugs	Modulation of tumor growth pathways	Glioma models	Tumor-homing properties; reduced tumor proliferation	Heterogeneity; safety concerns	[25,78,79]
**Dendritic cell EVs**	Immune cells	Immuno-modulatory cargo	Immune activation	Preclinical tumor models	Potential to enhance anti-tumor immunity	Limited CNS-specific validation	[84]
**Engineered EVs with targeting ligands**	Various	Small molecules/RNA	Receptor-mediated uptake	Glioma models	Improved targeting efficiency	Complex engineering requirements	[18,80,81,82,83]
**EV-mediated CRISPR delivery (experimental)**	Engineered cells	CRISPR/Cas components	Gene editing	Early-stage models	Proof-of-concept gene targeting	Delivery efficiency; safety	[86]
**Drug-loaded EVs (passive loading)**	Various	Chemo-therapeutics	Direct cytotoxicity	Glioma models	Improved drug stability and delivery	Variable loading efficiency	[78,79,84]
**Hybrid EV-nanoparticle systems**	Synthetic + EV hybrids	Drugs/RNA	Enhanced delivery platforms	Experimental systems	Increased stability and targeting	Regulatory complexity; scalability	[80,82]

## Data Availability

No new data were created or analyzed in this study.
